# Right Hepatic Artery Pseudoaneurysm Presenting As Upper Gastrointestinal Bleed: A Deadly Hit and Run Following Blunt Trauma

**DOI:** 10.7759/cureus.51232

**Published:** 2023-12-28

**Authors:** Eric S Atiku, Ifeoma Kwentoh, Shamon Gumbs, Brian Donaldson

**Affiliations:** 1 General Surgery, Columbia University College of Physicians and Surgeons, Harlem Hospital Center, New York, USA; 2 Internal Medicine, Columbia University College of Physicians and Surgeons, Harlem Hospital Center, New York, USA

**Keywords:** upper gastrointestinal (ugi) bleeding, blunt vascular trauma, right hepatic artery pseudoaneurysm, common hepatic artery pseudoaneurysm, hepatic trauma

## Abstract

Hepatic artery pseudoaneurysm (HAA) is a rare complication of blunt trauma, occurring in only 1% of patients. It is life-threatening with abysmal and atrocious outcomes if not recognized early and managed promptly. Other etiologies include transjugular-intrahepatic portosystemic shunt (TIPS), pancreatitis, cholecystectomy, and liver transplantation. We report a near-miss case in a 38-year-old woman following a motor vehicle accident. She sustained Grade III/IV liver laceration (>50% subcapsular hematoma), presenting with upper gastrointestinal bleeding (UGIB). Our patient was managed emergently intra-operatively, with hemostasis promptly achieved; however, she continued to bleed postoperatively, becoming hemodynamically unstable and unresponsive to both massive blood transfusions and high-dose proton pump inhibitors. Further imaging demonstrated HAA for which coil embolization was carried out by interventional radiology (IR).

## Introduction

Blunt injuries involving the porta hepatis are unusual occurrences with deadly outcomes if not diagnosed early [1]. Pseudoaneurysms are false aneurysms that may occur as a result of blunt trauma, it is a leakage of an injured artery into the tissues around the vessel with the formation of a new cavity outside the main artery [2]. The new lumen continues to communicate with the main artery with a risk of rupture due to increasing pressures built around and within the lumen [2,3]. In patients with blunt abdominal trauma, a very high degree of suspicion is employed at presentation, as these may not be apparent in the first imaging studies done in the emergency department. Bleeding due to leakage or even rupture may lead to hemoperitoneum, haemobilia, or gastrointestinal bleeding [4]. The diagnosis can be made by angiogram, contrast-enhanced CT, and Doppler ultrasounds [5]. Patients may have varying spectrum of presentation from asymptomatic to severe acute blood loss with hemoperitoneum. HAA can occur with varying degrees of liver lacerations. Liver injuries are graded based on the scale of the American Association for the Surgery of Trauma Organ Scaling Committee (AAST) from grades 1-4. Depending on the presentation and grade of liver injury, an open approach or gold standard by embolization may be the first line of management [6].

## Case presentation

A 38-year-old woman was brought into the emergency room after being struck by a motor vehicle. Her past medical history was notable for bipolar disorder but no hepatobiliary diseases. The primary survey noted a Glasgow Coma Scale (GCS) of 14, she appeared confused, combative, and uncooperative requiring sedation but not reporting any pain. Vitals were notable for tachycardia and hypotension with a blood pressure (BP) of 87/70 mm Hg. She had blunt trauma to the abdominopelvic area and abdominal distension, with multiple bruises to her extremities. A nasogastric tube was placed with output showing bloody fluid <200 ml, indicative of upper gastrointestinal bleed (UGIB). The initial chest X-ray was unremarkable. The pelvic X-ray showed an acute displaced right iliac crest fracture and a comminuted right acetabular fracture. The chest and pelvic X-rays were done in the trauma bay after the primary and secondary surveys as per Advanced Trauma Life Support guidelines prior to the patient leaving the trauma bay for more definitive studies.

**Figure 1:**
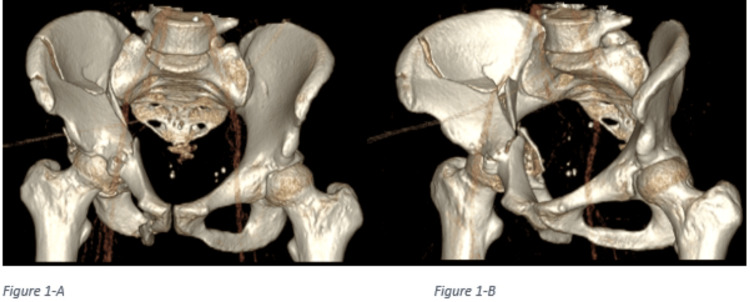
A: Acute displaced fracture of the body of the pubic bone + acute, comminuted, displaced fracture of the acetabulum with central protrusion of the right femoral head. B: Acute, comminuted fracture of the right iliac wing.

**Figure 2:**
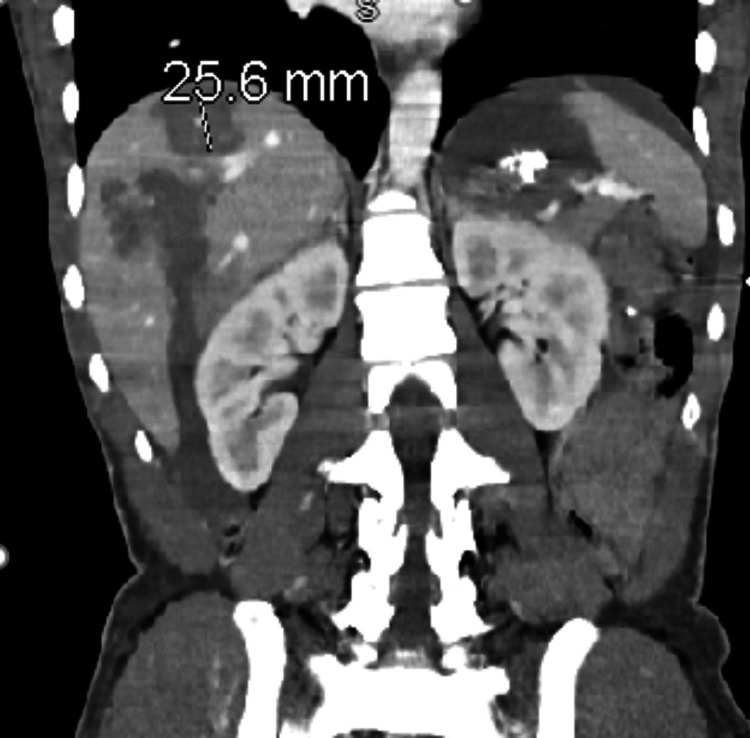
CT abdomen multiple lacerations in the right lobe of the liver largest measuring approximately 5.7 cm in depth

Given the mechanism of injury, the patient was "pan-scanned". Computed tomography (CT) scan of the head showed intracranial hemorrhage within the left frontoparietal lobe, left frontal subdural hematoma, left parietal epidural hematoma, and multiple scattered subarachnoid hemorrhage with a 7 mm midline shift and slight left uncal herniation. The CT abdomen and pelvis demonstrated an acute displaced fracture of the body of the pubic bone, acetabulum with central protrusion of the right femoral head, and right iliac wing (Figures 1A, 1B). The CT also showed multiple lacerations in the right lobe of the liver largest measuring approximately 5.7 cm in depth. There was an intraparenchymal hematoma measuring approximately 3.5 x 3.7 x 2.6 cm (Figure 2). A subcapsular hematoma involving greater than 50% of the surface area was noted. The findings were compatible with grade 3 to grade 4 traumatic injury of the liver.

She was emergently taken to the operation room, as she became hemodynamically unstable requiring a massive transfusion protocol for hemostasis of liver laceration. Intra-operatively, there was 750 ml of hemoperitoneum, large, non-expanding zone 3 hematoma, grade 4 liver laceration of segment 6, with no evidence of hollow viscus injury. Exploring the right upper quadrant, we removed the remaining clot without disturbing the clot within the grade 4 liver laceration of segment 6. There was a small amount of active bleeding from the laceration. We applied a flow seal to the laceration and packed over it with Surgicel ensuring adequate hemostasis. Next, we released the packs from the pelvis, we noted a large, non-expanding zone 3 hematoma, which was left untouched. The anterior stomach was normal, we entered the lesser sac and there was no hematoma, the posterior wall of the stomach appeared normal. The spleen was without apparent injury. There was a small amount of hematoma noted within the mesentery of the colon at the level of the splenic flexure, which was explored, there was no apparent injury to the colon. Hemostasis was achieved and the abdomen was closed. She was hemodynamically stabilized but transferred to the surgical intensive care unit for further care. The gastroenterology and hepatology team was consulted for the UGIB. High-dose proton pump (PPI) was recommended with a triple-phase CT abdomen.

**Figure 3:**
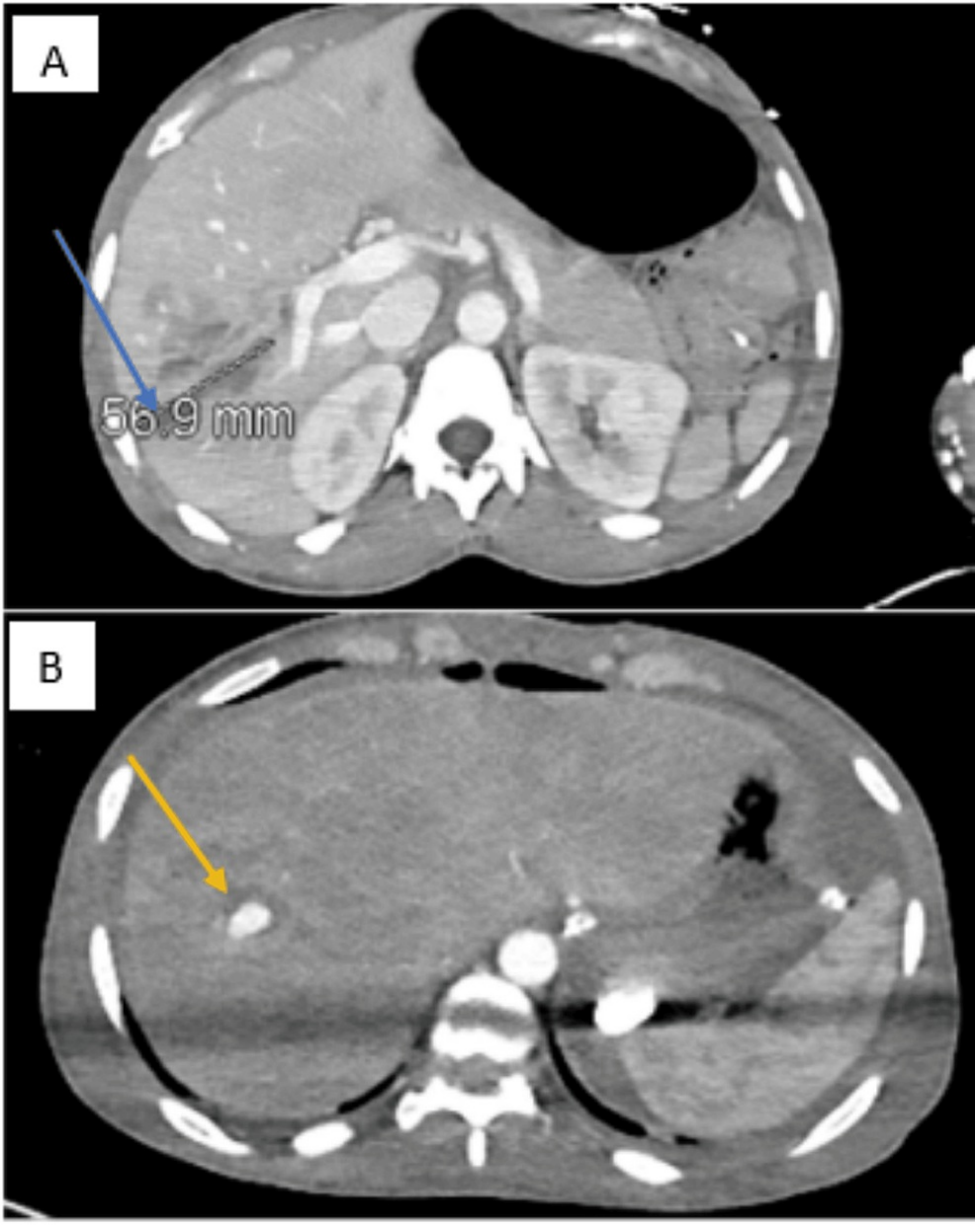
CT abdomen demonstrating hepatic artery aneurysm A- liver laceration (blue arrow); B- hepatic artery aneurysm (yellow arrow)

A repeat CT abdomen re-demonstrated multiple linear hepatic lacerations in the right hepatic lobe. A 4.2 x 1.9 cm elongated lobular contrast collection in an area of injury on arterial phase images, similar in morphology on portal venous images, likely representing a pseudoaneurysm (Figure 3).

**Figure 4:**
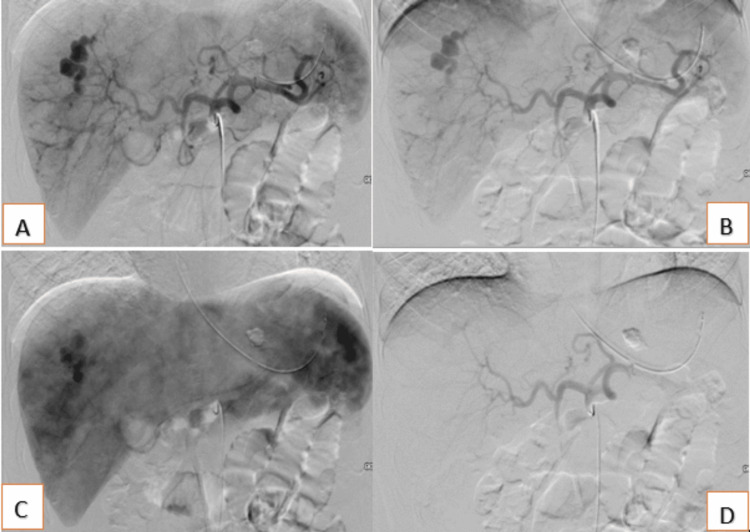
Percutaneous embolization of the hepatic pseudoaneurysm A+ B- interventional radiology- Angiogram Visceral Selective C+D- visceral angiography and probable embolization

Adjacent perihepatic hematoma along the inferior right hepatic lobe, more prominent compared to the earlier imaging, with air containing hemostatic Surgicel. Given the continued blood transfusion requirements despite surgical intervention, IR was consulted to evaluate for angioembolization. IR recommended emergency angiography and embolization (Video 1). She underwent a targeted hepatic angiogram and embolization (Figure 4).

**Video 1:** Intraoperative fluoroscopy images of the hepatic arteriogram and embolization (IR angiogram visceral selective) IR- interventional radiology

Initial celiac arteriogram and selective and super selective right hepatic arterial branch arteriograms showed a large pseudoaneurysm in the right hepatic lobe, corresponding with a known segment 7 laceration. Coil embolization of the subsegmental branch of the segment 7 hepatic artery using 2 mm Nester coils x 2 and 2 mm - 3 mm Tornado coils x 2. Completion of the right hepatic branch and celiac arteriograms show successful embolization with no further filling of the pseudoaneurysm (Video 1). Post-embolization arteriography of the segment 7 right hepatic artery branch and celiac artery demonstrated successful coil embolization of the subsegmental branch of the segment 7 right hepatic artery branch. Chemical venous thromboembolism prophylaxis and anticoagulation were held, so given the high risk for venous thromboembolism, an inferior vena cava filter was placed.

The right acetabular fracture was treated non-operatively by the placement of skeletal traction. The patient’s clinical course progressively improved, and she was subsequently discharged to an inpatient rehabilitation facility. She underwent inpatient physical therapy and rehabilitation with recovery of functional mobility. She was seen in the outpatient trauma surgery clinic one month later and was found to be recovering well. The patient currently follows up with the hepatobiliary and gastroenterology team post-discharge.

## Discussion

Visceral artery aneurysms and pseudoaneurysms are uncommon; however, they can be potentially disastrous pathologies. Hepatic artery aneurysms (HAA) are the second most common type of visceral artery aneurysms reported, although the actual incidence is unknown. The significance of timely diagnosis and management of these aneurysms is to prevent subsequent rupture into the peritoneal cavity, hepatobiliary, or gastrointestinal tract [6]. Injuries involving the porta hepatis due to blunt trauma are an unusual occurrence, however, when present, they have poor outcomes if there is a delay in diagnosis. Pseudoaneurysms are false aneurysms that may occur as a result of iatrogenic injury or trauma to the liver. A pseudoaneurysm is the leakage of an injured artery into the tissues around the vessel with the formation of a new cavity outside the main artery. The new lumen continues to communicate with the main artery with the risk of rupture due to increasing pressures built around and within the lumen [7].

HAA accounts for 25-80% of reported cases. The majority of pseudoaneurysms are symptomatic at presentation, thereby differing from true aneurysms, with gastrointestinal bleeding or haemobilia. In patients with blunt abdominal trauma, a very high degree of suspicion is employed at presentation, as these may not be apparent in the initial imaging studies. Bleeding due to leakage or even rupture may lead to hemoperitoneum, hemobilia, or gastrointestinal bleeding. Hepatic aneurysms may also come to attention due to erosion into the biliary tree, into the portal vein with the development of portal hypertension and its sequelae, or due to rupture into the retroperitoneal or peritoneal cavity [8]. A classic triad of right upper quadrant pain, jaundice, and upper gastrointestinal bleeding has been described (i.e. Quincke's triad), but this is present in only 25-30% of patients with hemobilia [8].

The Society for Vascular Surgery recommends that with any suspicion of HAA, the diagnosis be made by obtaining a CT angiogram of the abdomen [9]. Patients may have a varying spectrum of presentation, from asymptomatic to severe acute blood loss with hemoperitoneum. Liver injuries are graded based on the scale of the American Association for the Surgery of Trauma Organ Scaling Committee (AAST) from grades 1-4 [9]. Depending on the presentation and grade of liver injury, angioembolization is the usual first line management.

Our patient had massive hemoperitoneum with hemodynamic instability requiring exploratory laparotomy. During the exploration, a hematoma and clot over the liver injury were noted without active hemorrhage, so no further surgical intervention was indicated at that time. Postoperatively, the patient continued to be unresponsive to resuscitation and visceral angiography demonstrated a pseudoaneurysm, which was embolized (Figures 1-3). Areas for improvement would be performing primary repair of the liver laceration, which may have provided more hemostasis than hemostatic agents.

Both open and endovascular options exist for HAA repair. All retrospective case series have shown that the outcome for visceral artery aneurysms after open or endovascular repair yielded similar long-term results, but morbidity is significantly worse with open repair than with the endovascular approach.

Endovascular management of visceral pseudoaneurysms has proven to be effective, safe, and targeted in both elective and urgent cases [10]. This procedure conclusively has a high success rate with extremely few complications.

Intrahepatic aneurysms will require resection of the lobe in which the aneurysm is located. Given the significant comorbidities associated with liver resection, endovascular interventions have become the primary treatment modality for these intrahepatic lesions when feasible. Complications of embolization include hepatic ischemia, abscess, cholecystitis, and possible recanalization.

## Conclusions

It is crucial to recognize and address the potential complications of visceral aneurysms, including the high risk of rupture and mortality. Presentations vary from upper or lower acute gastrointestinal bleeding, including hemobilia. Prompt identification and intervention are critical for improving patient outcomes. This case highlights that the endovascular management of visceral pseudoaneurysm involved continued transfusion requirements despite surgical management. It also highlights maintaining a high index of suspicion, as despite surgical repair of hepatic lacerations, these complications can arise and be reliably treated if identified early.
